# Zina percutaneous screw fixation combined with endoscopic lumbar intervertebral fusion under intraoperative neuromonitoring: A case report

**DOI:** 10.1097/MD.0000000000024220

**Published:** 2021-03-19

**Authors:** Tong Yu, Jiu-Ping Wu, Jun Zhang, Hai-Chi Yu, Tian-Yang Yuan, De-Rui Xu, Zhi-He Yun, Tao He, Rui Liu, Qin-Yi Liu

**Affiliations:** Department of Orthopaedics, The Second Hospital of Jilin University, Changchun, Jilin Province, China.

**Keywords:** endoscopic, lumbar disc herniation, lumbar intervertebral fusion, minimally invasive, percutaneous

## Abstract

**Introduction:**

Traditional open discectomy and intervertebral fusion surgery is the common strategy for lumbar disc herniation (LDH). However, it has the disadvantages of long recovery time and severe paravertebral soft tissue injury. Zina percutaneous screw fixation combined with endoscopic lumbar intervertebral fusion (ZELIF), as a novel minimally invasive surgical technique for LDH, has the advantages in quicker recovery, less soft tissue destruction, shorter hospital stays and less pain. We report a novel technique of ZELIF under intraoperative neuromonitoring (INM) for the treatment of LDH.

**Patient concerns:**

A 51-year-old male presented to our hospital with left lower extremity pain and numbness for 1 year.

**Diagnosis:**

Lumbar disc herniation (LDH).

**Interventions:**

This patient was treated with Zina percutaneous screw fixation combined with endoscopic neural decompression, endplate preparation, and intervertebral fusion through Kambin's triangle. Each step of the operation was performed under INM.

**Outcomes:**

The follow-up period lasted 12 months; the hospitalization lasted 4 nights; the blood loss volume was 65 ml, and the time of operation was 266 min. INM showed no neurological damage during the surgery. No surgical complications, including neurological deterioration, cage migration, non-union, instrumentation failure or revision operation, were observed during the follow-up period. Visual Analogue Scale (VAS) score reduced from 7 to 1; the Oswestry Disability Index (ODI) decreased from 43 to 14; the EQ-5D score was 10 preoperatively and 15 at the final follow-up visit; the Physical Component Summary of the 36-Item Short Form Health Survey (SF-36) was 48 preoperatively and 49 at the last follow up visit; the SF-36 Mental Component Summary was 47 before surgery and decreased to 41 postoperatively.

**Conclusion:**

ZELIF under INM may represent a feasible, safe and effective alternative to endoscopic intervertebral fusion and percutaneous screw fixation, for decompressing the lumbar's exiting nerve root directly with minimal invasion in selected patients.

## 1 Introduction

In selected patients, spinal fusion is a procedure that has been proven to effectively improve the quality of life and significantly relieve pain.^[1,2]^ Various strategies can be used in lumbar intervertebral fusion (LIF), including posterior lumbar intervertebral fusion (PLIF), anterior lumbar intervertebral fusion (ALIF), oblique lateral lumbar intervertebral fusion (OLIF), transforaminal lumbar intervertebral fusion (TLIF), and direct lateral lumbar intervertebral fusion (DLIF). ALIF can provide adequate decompression of ventral dura, but it is not suitable for L4–5 and L5-S1 segments. PLIF can provide adequate decompression of the dorsal dural, but it is a relatively wide-open surgery and hence has the disadvantage of significant pain and long recovery time.

The application of tubular dilators for nerve root decompression, combined with percutaneous screws and specialized internal cages, makes it possible to replace open fusion surgery with minimally invasive surgery. However, a lot of intervertebral fusion surgeries minimally invasive still need a muscle-tissue incision to insert a tube. The technique of transforaminal endoscopy can achieve the minimally invasive decompression, and numerous endoscopic LIF strategies have been reported for LDH.^[3–7]^ However, various endoscopic LIF techniques have shortcomings, particularly in the placement of an established rigid bullet-shaped cage which is too large to pass through the endoscopic working channel.

Some surgical instruments (the square channel with C-shaped opening and the square channel clamp) were modified so that the endoscopic LIF could be completed easily. ZELIF has many advantages, such as allowing the traditional size cage insertion, safe nerve root decompression, a small volume of bleeding, short hospitalization time and rapid recovery. Here, a report on the steps, matters for attention, benefits, indications, and contraindications of the ZELIF technique is given.

## 2 Ethical approval

Patient has provided informed consent for publication of the case. This report was approved by the ethics committee of the Second Hospital of Jilin University, Changchun, China.

## 3 Case report

### 3.1 Patient characteristics

Examination revealed the significant lateral hypoesthesia of the left leg. The muscle strength of quadriceps femoris was at Grade V; the muscle strength of the left tibialis anterior was at Grade IV; the muscle strength of the left peroneus longus was at Grade III; the muscular tension of extremities, and the tendon reflex of the bilateral knees and bilateral Achilles were normal.

**Figure 1:**
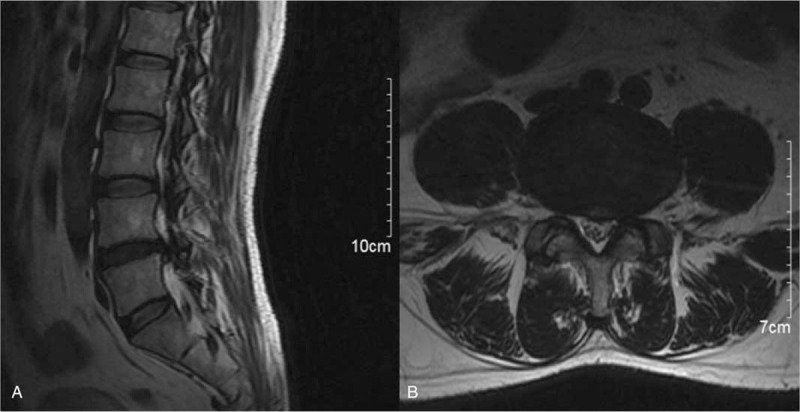
The sagittal (A) and axial (B) of MRI showed LDH (L4-5).

Magnetic resonance imaging (MRI) demonstrated LDH (L4-5) (Fig. 1A-B). The 3D CT of the lumbar spine showed straight lumbar curvature, lumbar osteoporosis, and L4-5 disc herniation. The lumbar vertebrae X-ray film displayed that the curvature of the lumbar spine became straight. Electromyography of lower extremities illustrated that the peroneus longus muscle and the tibialis anterior muscle were subject to neurogenic damage. The patient was diagnosed primarily with LDH (L4-5).

The surgical segment, operation time, hospitalization time, and surgical complications were recorded. VAS score, ODI score, SF-36 score, and EQ-5D were evaluated.

### 3.2 Anesthesia and INM techniques

General anesthesia with intubation was achieved using propofol (200 mg/kg), fentanyl (250 mg), and midazolam (2 mg). In addition, propofol (0.2–0.5 mg/kg per hour) was constantly infused for maintaining anesthesia. Short-acting muscle relaxants with succinylcholine (1 mg/kg) were only provided during induction and intubation. The intraoperative nerve monitoring technique from Yu et al.^[8,9]^ was applied in this surgery.

### 3.3 Operative technique

**Figure 2:**
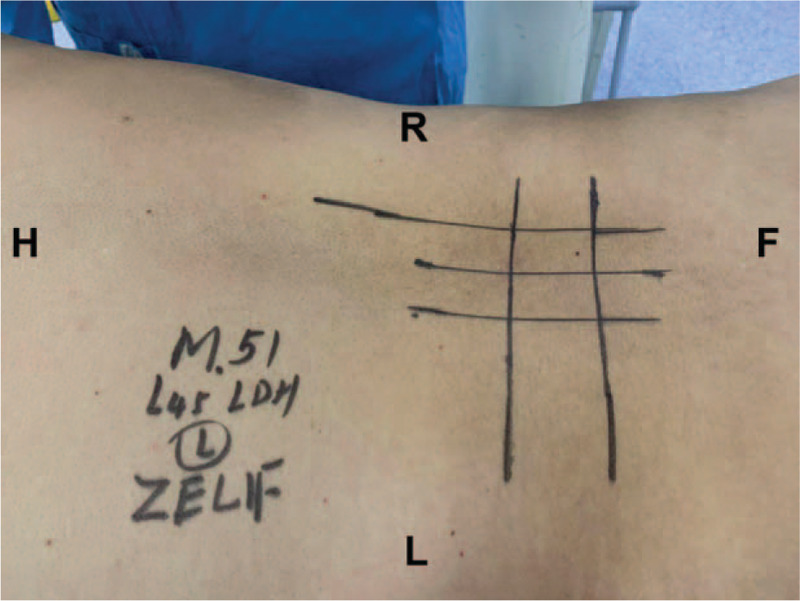
The patient was placed in a prone position.

**Figure 3:**
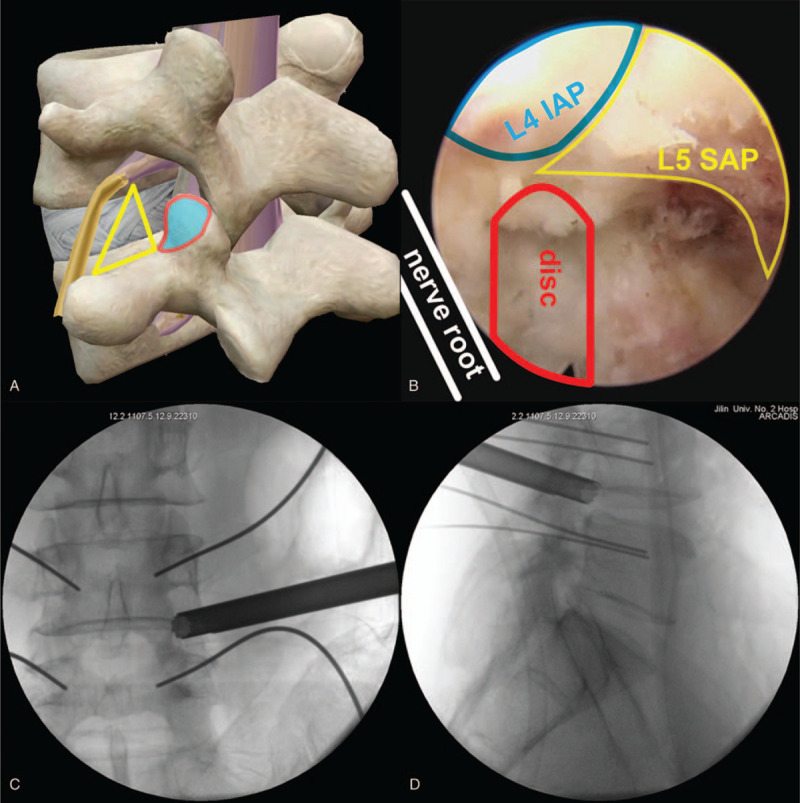
The yellow triangle represents Kambin's triangle and the bone in the red circle is part of the L5 that needs to be resected (A), the blue, yellow and red regions represent L4 inferior articular process and L5 superior articular process after partial resection (B), anteroposterior (C) and lateral (D) radiographs of the L5 superior articular process arthroplasty.

**Figure 4:**
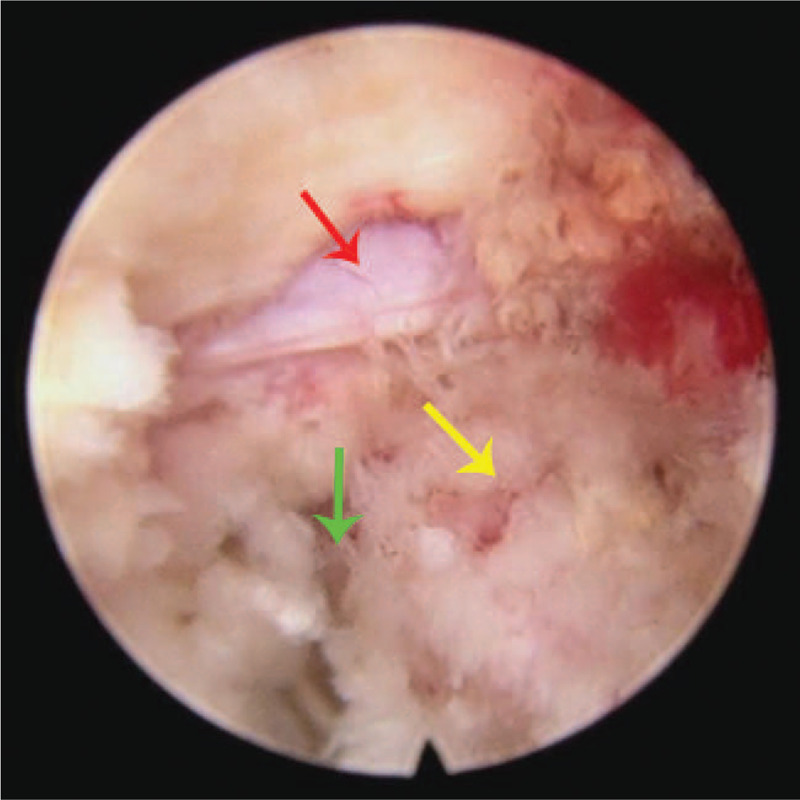
Nerve root decompression was conducted under endoscopic. The red arrow represents the nerve root; the yellow arrow represents the endplate and the green arrow represents the intervertebral space after decompression.

**Figure 5:**
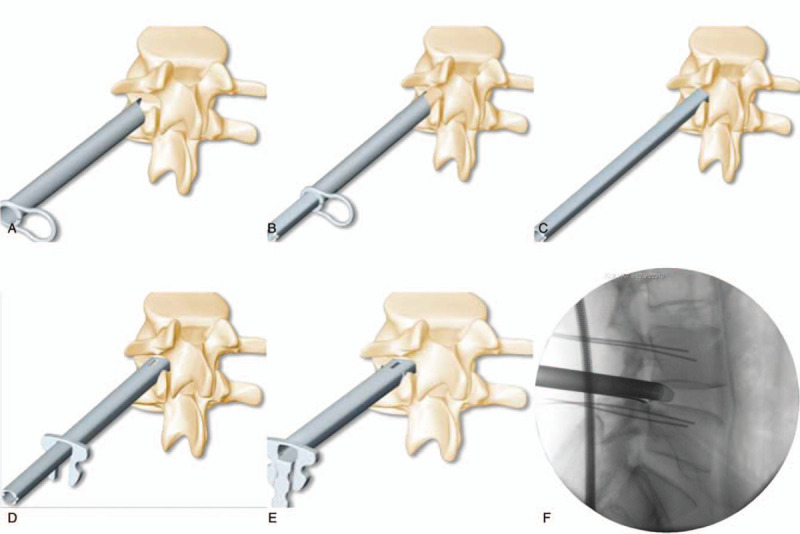
Round canula implantation (A), square canula insertion (B), round canula (C) was taken out); the square channel with C-shaped opening (D) was inserted; the square channel clamp (E) was installed; the lateral radiograph (F) showed that the establishment of the channel was completed successfully.

**Figure 6:**
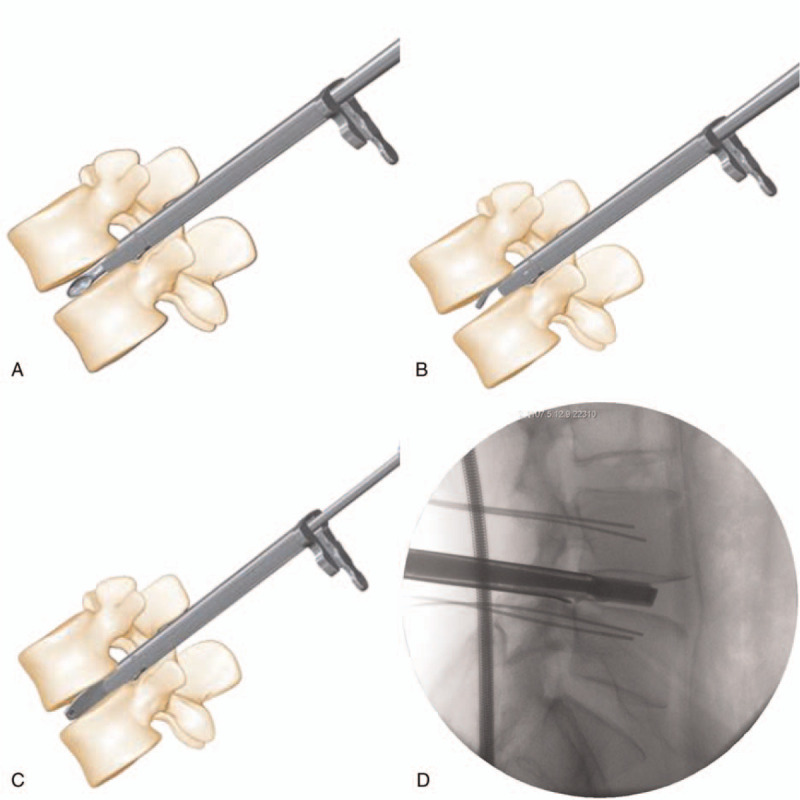
The intervertebral disc tissue was removed with a scraping spoon (A), a triangular scraping spoon (B), and a scraper (C and D).

**Figure 7:**
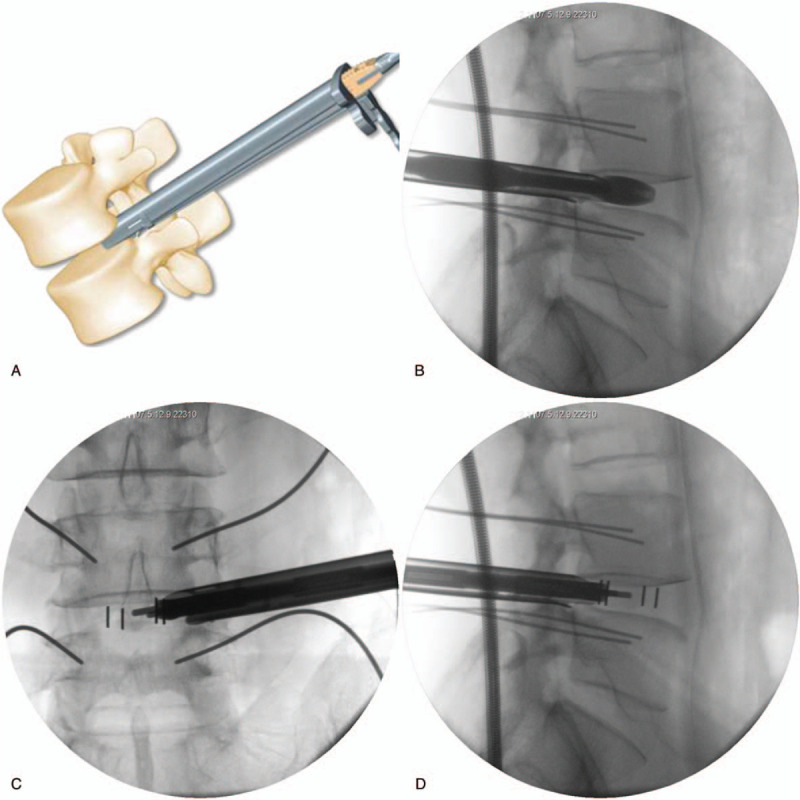
According to the test model (A and B), the appropriate size of the cage was selected, and the position of the cage was examined by anteroposterior (C) and lateral (D) radiographs.

**Figure 8:**
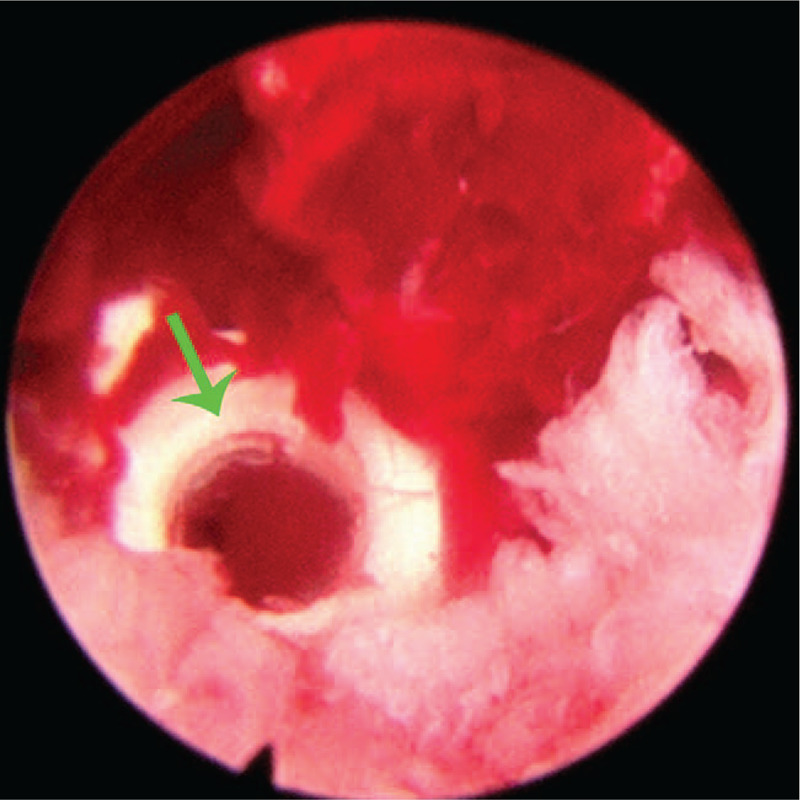
Endoscopic observation of the cage that indicated by blue arrows.

**Figure 9:**
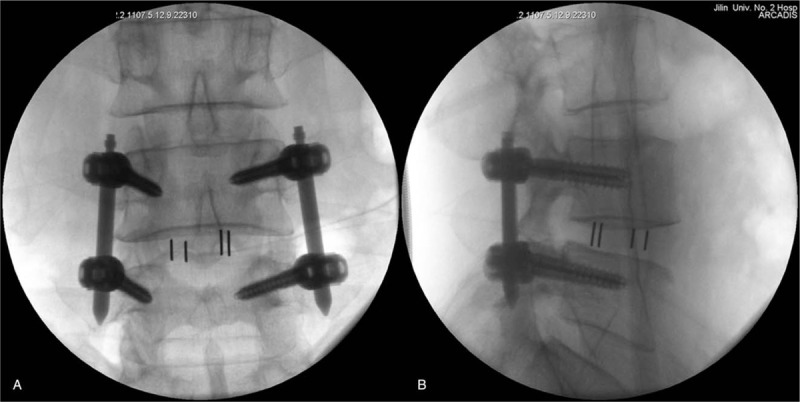
Anteroposterior (A) and lateral (B) radiographs were taken postoperatively and showed that the Zina percutaneous pedicle screws and the cage were in a good position.

(1) The patient was positioned prone on an operating table after general anesthesia (Fig. 2).
(2) The needle of Zina percutaneous pedicle screw (Shanghai Sanyou Medical Devices Co., Ltd. China) was inserted. If the intervertebral space was too small, it should be distracted; if the intervertebral space was not narrow, it should not be distracted.
(3) Endoscope implantation (Medical Intervertebral Endoscopic Surgery System, Shenzhen Shenzhou Medical Equipment Co., Ltd. China) was implemented. The endoscopic incision was located at 2to 3 transverse fingers outside the pedicle and 50to 60 ° from the horizontal plane. The endoscopic saw was used to remove part of the superior articular process of L5 vertebral body to enlarge the intervertebral foramen (Fig. 3A–D) and complete the arthroplasty of the superior articular process of L5 vertebral body. **Note:** the superior articular process should not be too large in case of massive bleeding.
(4) Nerve root decompression was performed under endoscopic (Fig. 4), and all endoscopic tools were removed after decompression.
(5) ZELIF tools (Shanghai Sanyou Medical Devices Co., Ltd. China) (Fig. 5A–F) were installed. The key parts of ZELIF tools are the expandable square channel with C-shaped opening and the square channel clamp, the main function of which is to simplify the installation of the cage device. ZELIF incision was performed using the same method as that of endoscope incision. The guide needle was placed under fluoroscopy. The direction of the guide needle was parallel to the vertebral endplate on the lateral radiograph, and the tip of the needle was located in the center of the intervertebral space on the anteroposterior radiograph. The square canula was inserted into the intervertebral space along the direction of the guide needle, and then a square channel with C-shaped opening was inserted into the intervertebral space along the direction of the square sleeve; the square canula and the guide needle were taken out; at last the square channel clamp was installed to complete the establishment of the channel. **Note:** I. The square channel must be placed in the Kambin's triangle^[10]^ to avoid nerve root injury. II. The functional status of the nerve root should be monitored during operation.
(6) Intervertebral space management (Fig. 6A–D). The intervertebral disc tissue was removed with pliers, a scraping spoon, a triangular scraping spoon, a scraper, and a reamer.
(7) According to the test model, the cage with an appropriate size was selected and then inserted **(**Fig. 7A–D). The position of the cage was confirmed by fluoroscopy, and the square channel was taken out.
(8) Endoscopic exploration of the nerve root and the cage (Fig. 8).
(9) A Zina screw was inserted and a titanium rod was installed (Fig. 9A–B).

### 3.4 Outcomes

The follow-up period lasted 12 months; the hospitalization lasted 4 nights. The blood loss volume was 65 ml, and the time of operation was 266 min. INM showed no neurological damage during surgery. No surgical complications, such as neurological deterioration, cage migration or non-union, were found during the follow-up period (Table 1). The VAS score reduced from 7 to 1; the ODI score decreased from 43 to 14; the EQ-5D score was 10 preoperatively and 15 at the final follow-up visit; the SF-36 Physical Component Summary was 48 preoperatively and 49 at the last follow up visit; and the SF-36 Mental Component Summary was 47 before surgery and decreased to 41 postoperatively (Table 2).

**Table 1:** The basic information of the patient.

Parameter	Case
Age (year)	51
Gender (male/female)	male
Lesion segment	L4-5 (left)
Operation time (min)	266
Hospitalization time (night)	4
Blood loss (ml)	65
Infection	none
Recurrence of LDH	none
INM events	none
Surgical complication	none
Follow up (month)	6

INM = intraoperative neuromonitoring, LDH = lumbar disc herniation.

**Table 2:** Evaluation parameters were collected preoperatively and postoperatively.

	ODI score	SF-36 score (P)	SF-36 score (M)	VAS score	EQ-5D
Preoperative	43	48	47	7	10
Postoperative	14	49	41	1	15

EQ-5D = EuroQol five dimensions questionnaire, ODI = Oswestry Disability Index, SF-36 score (M) = the 36-Item Short Form Health Survey (SF-36) Mental Component Summary, SF-36 score (P) = the 36-Item Short Form Health Survey (SF-36) Physical Component Summary, VAS = Visual Analogue Scale.

## 4 Discussion

Compared with other surgical disciplines including open surgery and sports medicine in orthopedics, endoscopic minimally invasive surgery has been considered to be a sensible method that satisfies the demands of the quicker recovery for LDH in recent years.^[7]^ Minimally invasive spinal surgery is gaining popularity because its several advantages, including restoring functions while preserving normal anatomy, minimizing hospitalization and complications associated with extensive open procedures, and helping elderly patients return to active premorbid status as early as possible.^[6]^ Endoscopic lumbar intervertebral fusion has been reported. Since the incidence of complications is as high as 36%, it is not recommended by Jacquot et al.^[5]^ However, ZWLIF technique can finish lumbar intervertebral fusion and percutaneous screw fixation safely without technical limitations. To the best of our knowledge, no study on ZELIF has been reported yet. Thus, we intended to share a relevant technical note.

Numerous prevenient studies have discussed the anatomical structure of intervertebral foramina in the lumbar spine, so as to measure the maximum working channel space suitable for undergoing endoscopic discectomy manipulations.^[11–14]^ In 1995, Mirkovic et al.^[13]^ reported the size of the safe edge for a channel in the intervertebral foramen. In 2005, Min et al.^[12]^ demonstrated that the average distance between the superior articulating process and the exiting nerve root was 11.6 mm. In 2016, from a cadaveric study, Hardenbrook M et al.^[15]^ reported a relatively large area in the lumbar intervertebral foramen, called Kambin's triangle. Therefore, we believe that the ZELIF channel is safe to be installed through Kambin's triangle, and this idea has been agreed by Ozer et al.^[10]^ Although it is theoretically safe, INM is carried out to prevent nerve injury.

Indications of this technique include

(1) LDH with segmental instability,
(2) lumbar spinal stenosis with segmental instability and
(3) lumbar spondylolisthesis of lower than Meyerding grade II.

Contraindications mainly include

(1) L5-S1 lumbar disc herniation due to the high ilium which affects the installation of the channel,
(2) variation of the nerve root,
(3) lumbar spondylolisthesis of higher than Meyerding grade III, and
(4) severe central canal stenosis.

Advantages of ZELIF include shorter operation time, less blood loss, shorter hospital stays, a very low risk for pulmonary embolism, less soft tissue destruction, less pain, quicker restoring the function of standing and walking, and no need for drain postoperatively.^[5]^ In addition, the decompression of dura and the nerve root was performed under endoscope and INM, avoiding nerve injury and improving the safety of operation.

Literature reports suggest that smaller fusion cages must be used when endoscope-guided intrabody cages are placed, because traditional fusion cages are too large to pass through working channels.^[5,16]^ However, the undersized cage may directly cause cage migration into the intervertebral foramen or spinal canal, thus leading to neurological compromise, failure of fixation, or even revision operation.^[17,18]^ The ZELIF technology uses a specially designed C-shaped open channel, which can realize the installation of conventional cages under fluoroscopy without size reduction. In this study, cage migration was not observed during 1 month of the follow-up, and we attribute this positive result to the ZELIF technique.

INM has been commonly utilized to avoid nerve damage in spinal surgery,^[9,19]^ and considered by many scholars as a reliable method to avoid nerve injury.^[9,19,20]^ Even if there are no neuromonitoring events during the operation, we believe that it is necessary to monitor the functional state of the nerve root, especially in the process of channel installation. We suggest that once abnormal nerve monitoring is found, the installation direction of the channel should be adjusted in time.

The following points should be paid attention to during the operation: (1) After the establishment of the channel, it is suggested that the assistant should fix the channel by hand to avoid it sliding out due to improper operation; (2) If the channel is taken out accidentally, please re-enter the guide wire and re-install the channel. Blind direct installation of the channel is forbidden; (3) Careful examination of the patients should be performed preoperatively, and nerve root variation is not suitable for ZELIF; (4) INM is recommended during all surgical steps.

ZELIF under INM may represent a feasible, safe and effective alternative to endoscopic intervertebral fusion and percutaneous screw fixation with minimal invasive in selected patients for decompressing the exiting nerve root of the lumbar directly.

## Acknowledgments

We gratefully acknowledge the cooperation of the doctors and nurses in the operating room.

## Author contributions

**Conceptualization:** Rui Liu.

**Methodology:** Jiuping Wu, Jun Zhang, Haichi Yu, Tianyang Yuan, Tao He.

**Project administration:** Jiuping Wu, Haichi Yu.

**Resources:** Zhihe Yun.

**Supervision:** Qinyi Liu.

**Validation:** Tong Yu, Tao He.

**Writing – original draft:** Tong Yu, Jun Zhang, Derui Xu.

**Writing – review & editing:** Qinyi Liu.
